# FCGRs Polymorphisms and Response to Trastuzumab in Patients With HER2-Positive Breast Cancer: Far From Predictive Value?

**DOI:** 10.14740/wjon934w

**Published:** 2015-10-26

**Authors:** Andrea Botticelli, Federica Mazzuca, Marina Borro, Eva Mazzotti, Marco La Torre, Adriana Bonifacino, Francesca Romana Ciabatta, Giovanna Gentile, Chiara Maddalena, Maurizio Simmaco, Paolo Marchetti

**Affiliations:** a Department of Molecular and Clinical Medicine, “Sapienza” University of Rome, Sant’Andrea Hospital, Rome, Italy; b Department of Neurosciences, Mental Health and Sensory Organs (NESMOS), Sapienza University of Rome - Advanced Molecular Diagnostics Unit (DiMA), Sant’Andrea Hospital, Rome, Italy; c Department of General Surgery, Sant’Andrea Hospital, Rome, Italy

**Keywords:** Breast cancer, FCGR, Polymorphisms, Trastuzumab

## Abstract

**Background:**

The aim of the study was to validate the association between the Arg166His polymorphisms of the Fc immunoglobulin receptor 2A (FCGR2A) and the Val212Phe of FCGR3A and pathological clinical response (pCR) to trastuzumab in HER2-positive breast cancer patients.

**Methods:**

Polymorphisms were characterized by pyrosequencing in 26 patients with ductal histotype breast cancer in a neoadjuvant setting and genotype association with pCR was analyzed.

**Results:**

No association was found between the FCGR3A Val212Phe polymorphisms and pCR. In contrast, the FCGR2A GG genotype (Arg allele) was found to be positively associated with pCR (P = 0.012).

**Conclusions:**

Our results do not support previously reported data on the effect of polymorphisms in immunoglobulin Fc receptors upon response to trastuzumab therapy.

## Introduction

Trastuzumab (trade name, Herceptin^®^) is a humanized IgG1 monoclonal antibody (mAb) that targets the human epidermal growth factor receptor family member HER2 and that has shown efficacy in the treatment of HER2 over-expressing breast and gastric cancers [1]. Although it represents a targeted therapy [2], only 25-30% of treated patients have an objective response. Hence, a biomarker allowing preemptive detection of responders and non-responders is strongly needed to identify patients who are likely to benefit from trastuzumab treatment.

The mechanisms of action of therapeutic mAbs are not fully elucidated, but the antibody-dependent cellular cytotoxicity (ADCC) seems to have a main role in their anti-tumor activity [3]. ADCC is mediated by the ability of specific immunoglobulins G (IgG) to crosslink the tumor cell with a cytotoxic immune cell (natural killer, monocyte or macrophage). The tumor-specific cell antigen is bound by the Fab fragment of the therapeutic mAb, while its Fc fragment recognizes specific receptors on the immune cells, the Fc gamma receptor (FCGR). The binding of FCGRs to the Fc region of immunoglobulins mediates a variety of immune functions, such as antigen presentation, clearance of immune complexes, phagocytosis of pathogens, degranulation, ADCC and cytokine production [4-6].

In human, the three classes of FCGRs (FCGR1/CD64, FCGR2/CD32 and FCGR3/CD16), encoded by specific genes (FCGR1A, -B, -C; FCGR2A, -B1, -B2, -B3, -C; FCGR3A, -B), present different affinity for different IgG subclasses and cell type-specific expression patterns. The IgG1 subclass binds the FCGR1s with high affinity but can bind also FCG2Rs and FCG3Rs with low affinity [7]. Thus, the IgG1 mAbs (used in oncology and hematology) have the possibility to engage all of the FcgRs to strengthen the recruitment of immune cells on the target tumor cells [8, 9]. This consideration leads to study of the relationship between the polymorphisms in FCGR genes and the response to treatment with monoclonal antibodies. The most studied polymorphisms are the His131Arg in the FCGR2A gene and the Val212Phe in the FCGR3A gene. The FCGR2A His131Arg (rs1801274, A>G) leads to the substitution of a histidine with an arginine residue. The His and the Arg alleles are also known, respectively, as low responder (LR) and high responder (HR) alleles, according to the weak or strong reactivity with mouse IgG1 (mIgG1). The LR and HR alleles also differ significantly in their ability to bind human IgG2, with a more efficient binding of the LR allele compared to the HR allele.

The FCGR3A Val212Phe (rs396991, T>G) leads to the substitution of a valine with a phenylalanine residue. The Val variant shows a stronger binding affinity for all the human IgG subclasses [10-12].

The different affinity for IgG binding of different alleles of FC receptors is postulated to affect the strength of response to trastuzumab treatment, and thus its efficacy. Though, association studies investigating FCGRs genotypes and clinical response to trastuzumab lead to contrasting results [2, 13-15]. This study was conceived to assess the effects of FCGR2A and 3A polymorphisms on pathological complete response (pCR) in breast cancer patients treated with trastuzumab-based neoadjuvant.

## Materials and Methods

### Patient population

Our institutional ethics committee approved this study, and written informed consent was obtained from each patients. A total of 26 consecutive women with histological confirmed ductal breast cancer, and HER2 positivity (IHC 3+ or positive FISH) were enrolled from May 2012 through September 2013. Mean age was 49.5 years (range 37 - 79 years).

Patients agreed to the collection and testing of their blood and were willing and able to provide approximately 10 mL blood draw.

They received epirubicin-cyclophosphamide (60/600 mg/mq 3 weeks) and taxane (paclitaxel 80 mg/mq weekly IV × 12 or docetaxel 75 mg/mq 3 weeks IV × 4) - trastuzumab (4 mg/kg followed by 2 mg/kg × 12 or 8 mg/kg followed by 6 mg/kg 3 weeks × 4) based chemotherapy in neoadjuvant setting.

The pCR was defined as “no detectable invasive residual cancer in the breast or lymph nodes by histopathology”. Toxicities were registered during every medical examination and they were classified using Common Terminology Criteria for Adverse Events v4.

### Inclusion criteria

Inclusion criteria include histological confirmed ductal breast cancer, locally advanced or inflammatory disease, HER2 positivity (IHC 3+ or positive FISH), measurable disease, chemotherapy susceptibility, age ≥ 18 years, Eastern Cooperative Oncology Group performance status of 2 or less, and to be able to undergo standard of care imaging studies (same imaging/staging modality being used at each evaluation).

### Genotyping

Genomic DNA was isolated from blood samples using the X-tractor Gene system (Corbett Life Science, Australia).

Reference sequence for FCGR2A and FCGR3A gene was obtained from NCBI GenBank database (http://www.ncbi.nlm.nih.gov/).

Genotyping was performed by pyrosequencing technology, using the Pyrosequencer PyroMark ID system (Qiagen) according to manufacturers’ directions. Both the amplification and the sequencing primers were obtained by the PSQ Assay Design software (Biotage AB and Biosystems, Uppsala, Sweden). Primers sequences for FCGR2A were: forward, AAAATCCCAGAAATTCTC; reverse, ATACCTTGGACAGTGATG; sequencing, AAGGTGGGATCCAAA.

Primers sequences for FCGR3A were: forward, AAAGGCAGGAAGTATTTT; reverse, TGTCTCACCTTGAGTGAT; sequencing, TTCTGCAGGGGGCTT.

PCR reaction was performed in a final volume of 25 μL containing 70 ng of genomic DNA, 10 pmol of each primer, 0.2 mM dNTPs, PCR buffer and 0.75 U of Taq DNA polymerase (Takara Bio Inc., Otsu, Japan), 2 mM MgCl_2_. PCR conditions were: 95 °C for 3 min; 40 cycles: 95 °C for 20 s, 50 °C for 20 s, 72 °C for 20 s; 72 °C for 5 min.

### Statistics

Test for deviation from the Hardy-Weinberg equilibrium was performed using the online tool Finetti’s Program (https://ihg.gsf.de/cgi-bin/hw/hwa1.pl). Genotypes distribution was assessed by Fisher’s exact test, considering a P < 0.05 significant.

## Results

All 26 patients had a G3 histological grade cancer, and 12 of 26 patients (46.2%) presented a complete response to neoadjuvant trastuzumab therapy. The analyzed polymorphisms did not deviate from the Hardy-Weinberg equilibrium. Genotypes’ distributions according to pCR are reported in Table 1. A statistically significant association was found between FCGR2A Arg/Arg (genotype G/G) genotype and pCR (P = 0.012), whereas no association was detected for the FCGR3A polymorphism.

**Table 1:** Distribution of Genotypes in Responders (Yes) and Non-Responders (No) to Trastuzumab

	pCR		P-value
	Yes (N = 12)	No (N = 14)	
FCGR2A His131Arg (A>G)			
AA	3 (25.0)	7 (50.0)	
AG	3 (25.0)	7 (50.0)	
GG	6 (50.0)	0 (0.0)	0.012
FCGR3A Val212Phe (T>G)			
TT	3 (25.0)	4 (28.5)	
TG	8 (66.7)	7 (50.0)	
GG	1 (8.3)	3 (21.5)	0.590

pCR: pathological clinical response.

## Discussion

Trastuzumab is a humanized anti-HER2 IgG1 antibody that has shown efficacy in HER2-positive breast cancer. Although it represents a targeted therapy, only 25-30% of metastatic HER2-positive breast cancer patients have an objective response to this treatment and only 30% of patients have a complete pathological response in neoadjuvant setting [9]. The therapeutic effect of trastuzumab is due to its binding to the receptors of the Fc fragment of immunoglobulins and activation of downstream cellular pathways, mediating the immune response against tumor cells.

Our results are surprising since they contrast with previous studies, in particular with regard to the FCGR2A His131Arg polymorphism.

Musolino et al [2] reported that, in breast cancer samples, the trastuzumab-mediated cytotoxicity is higher for the genotypes FCGR2A His/His and FCGR3A Val/Val compared to other genotypes. Further, in a sample of 15 operable and 35 metastatic HER2-positive breast cancer patients, FCGR2A His/His predicted the response to trastuzumab in neoadjuvant chemotherapy and in metastatic setting, while FCGR3A Val/Val did not [13]. In another sample of 54 HER2/neu-amplified metastatic breast cancer patients that received trastuzumab, those with FCGR3A Val/Val reported a better objective response rate (ORR) and progression free survival (PFS); only a trend of significance was found for FCGR2A His/His [2]. Finally, Hurvitz and colleagues did not find any association between FCGR3A and FCGR2A polymorphisms and disease free survival [14], in 1,189 patients treated with trastuzumab in adjuvant setting. The same negative result has been reported by Kim and colleagues in 57 HER2-positive metastatic breast cancer patients [15].

Hence, while our study confirms the lack of association between the FCGR3A Phe158Val and trastuzumab efficacy, we describe for the first time a completely reversed effect of the FCGR2A His131Arg, since we found that 100% of patients’ carriers of the homozygous FCGR2A Arg allele developed complete pathological response to treatment.

Obviously our study is really limited by the small number of patients; nonetheless it suggests that the effective predictive power and clinical utility of FCGRs polymorphisms remain masked by underling confounding factors.

Differences in clinical settings and clinical outcome measures are the main factors which imply potential discordance of results. However, the failure in validation of results among studies should also lead to better investigation of the molecular and cellular mechanisms by which trastuzumab acts. Indeed, Salmon et al [16], Parren et al [17] and more recently, Omar et al [18] showed that there are not significant differences in the binding index towards human IgG1 and the His and Arg alleles of the FCGR2A. Thus, it turns doubtful that the functional effect of this polymorphism, if real, is truly mediated by a different affinity for the receptor, and alternative mechanistic explanations should be tempted.

In conclusion, the potential of FCGRs polymorphisms as predictive markers of trastuzumab response is still controversial and future studies should carefully consider consistency of clinical setting and outcome measurement.
